# Huaier Aqueous Extract Induces Hepatocellular Carcinoma Cells Arrest in S Phase via JNK Signaling Pathway

**DOI:** 10.1155/2015/171356

**Published:** 2015-06-28

**Authors:** Chengshuo Zhang, Jialin Zhang, Xin Li, Ning Sun, Rui Yu, Bochao Zhao, Dongyang Yu, Ying Cheng, Yongfeng Liu

**Affiliations:** ^1^Department of Hepatobiliary Surgery and Unit of Organ Transplantation, First Hospital of China Medical University, 155 Nanjingbei Street, Heping District, Shenyang 110001, China; ^2^Department of General Surgery, Fourth Hospital of China Medical University, 4 Chongshandong Road, Huanggu District, Shenyang 110032, China; ^3^Department of Gastrointestinal Surgery, First Hospital of China Medical University, 155 Nanjingbei Street, Heping District, Shenyang 110001, China

## Abstract

Huaier aqueous extract, the main active constituent of Huaier proteoglycan, has antihepatocarcinoma activity in experimental and clinical settings. However, the potential and associated antihepatoma mechanisms of Huaier extract are not yet fully understood. Therefore, in this study, we aimed to elucidate the inhibitory proliferation effect of Huaier extract on apoptosis and cycle of HepG2 and Bel-7402 cells. Our data demonstrated that incubation with Huaier extract resulted in a marked decrease in cell viability dose-dependently. Flow cytometric analysis showed that a 48 h treatment of Huaier extract caused cell apoptosis. Typical apoptotic nucleus alterations were observed with fluorescence microscope after Hoechst staining. Immunoblot analysis further demonstrated that Huaier extract activated caspase 3 and PARP. Additionally, Huaier extract inhibited the activity of p-ERK, p-p38, and p-JNK in terms of MAPK. Furthermore, Huaier extract induced HCC cells arrest in S phase and decreased the cycle related protein expression of *β*-catenin and cyclin D1. Studies with JNK specific inhibitor, SP600125, showed that Huaier extract induced S phase arrest and decreased *β*-catenin and cyclin D1 expression via JNK signaling pathway. In conclusion, we verify that Huaier extract causes cell apoptosis and induces hepatocellular carcinoma cells arrest in S phase via JNK pathway, which advances our understanding on the molecular mechanisms of Huaier extract in hepatocarcinoma management.

## 1. Introduction

Hepatocellular carcinoma (HCC) is the fifth most frequent malignancy and the second leading fatal disease worldwide [1]. There are several canonical strategies for hepatocellular carcinoma treatment, including surgical resection, local ablation, liver transplantation, radiotherapy, chemotherapy, and molecular targeted therapy [2–4]. However, these treatments cause serious side effects sometimes; they are not applicable to all of the patients and demonstrate to be of limited efficacy [5, 6]. Therefore, identification of novel antitumor agents from natural products with better effectiveness is an alternative choice for management of hepatocellular carcinoma.

In recent years, many Chinese herbs have been gradually discovered to be potential sources of antitumor drugs for its role in killing tumor cells less intensively and more naturally [7, 8].* Trametes robiniophila* Murr. (Huaier) has been applied in traditional Chinese medicine for approximately 1600 years [9]; however, its antitumor properties are found and used as a complementary therapy only in recent decades. The main effective ingredient of this officinal fungi has been identified as proteoglycan which contains 41.53% polysaccharides, 12.93% amino acids, and 8.72% water [10]. A number of studies have demonstrated that Huaier extract inhibited proliferation and induced apoptosis in pulmonary cancer, breast cancer, melanoma, and colorectal cancer [11–14]. In addition, Huaier extract has also been indicated as a suppressant in angiogenesis and cell motility of ovarian cancer [6, 15]. The accumulating evidences have demonstrated that Huaier extract dose-dependently inhibited the proliferation, adhesion, migration, invasion, and angiogenesis and induced apoptosis of hepatoma cells [16, 17]. However, the underlying molecular mechanisms of Huaier extract activities in hepatocellular carcinoma cells are not yet fully understood.

Cell cycle deregulation, resulting in uncontrolled cell proliferation, is one of the most common alterations that occur during tumor development. Therefore, cell cycle arrest is considered to be an effective strategy for eliminating cancer cells [18]. Two major checkpoints, one at the G1/S transition and the other at the G2/M transition, regulate the cell cycle and, therefore, the modulated expression of cell cycle regulatory molecules on antiproliferation has been investigated in numerous cell types [19]. A general critical event associated with DNA damage is the activation of cell cycle checkpoints and cycling and cyclic-dependent kinases (cdks) are evolutionarily conserved proteins that are essential for cell cycle control [20]. Distinct pairs of cyclins and cdks regulate the progression through the various stages of the cell cycle; cdk activity is regulated by cyclins, which bind to and activate cdks [21]. Among these cyclins, cyclin D1 is regarded as an oncogene and is a major driver of multiple types of human tumors including breast and squamous cell cancers, B-cell lymphoma, myeloma, and parathyroid adenoma [22]. In addition to cyclin D1 and its upstream effector *β*-catenin [23], the mitogen-activated protein kinase (MAPK) superfamily members are also associated with increased migration, invasion, proliferation, survival, and apoptosis, thus serving different roles in cellular responses [23]. ERK1/2, p38 MAPK, and JNK/SAPK have been reported to play a central role in the regulation of *β*-catenin and cyclin D1 expression [24].

Little is known about the molecular mechanisms responsible for the proliferative properties of Huaier extract, and no studies have investigated the potential role of Huaier in cell proliferation of human HCC cells. Understanding the mechanism of action of Huaier extract should provide useful information for their possible application in cancer therapy and cancer prevention. Therefore, in this study we mainly aim to explore the antiproliferation mechanisms of Huaier extract in HCC cells.

## 2. Materials and Methods

### 2.1. Preparation of Huaier Aqueous Extract

Huaier electuary ointment was a gift from Gaitianli Pharmaceutical Co., Ltd. (Qidong, Jiangsu, China). Two grams of the electuary ointment was dissolved in 20 mL of complete medium and was sterilized with 0.22 *μ*m filter to get the 100 mg/mL stock solution for long storage at −20°C [25].

### 2.2. Cell Culture

HCC cell lines, including HepG2 and Bel-7402, were grown in RPMI 1640 medium (Gibco, USA) in the presence of 10% fetal bovine serum (Gibco, USA) and incubated in a humidified atmosphere containing 5% CO_2_ at 37°C. Immortalized normal liver epithelial cells, THLE-3, were maintained in Dulbecco's modified Eagle's medium (DMEM) (Invitrogen, Carlsbad, CA) supplemented with 100 U/mL penicillin and 100 *μ*g/mL streptomycin in the presence of 10% fetal bovine serum and incubated in a humidified atmosphere containing 5% CO_2_ at 37°C.

### 2.3. Cell Proliferation Assay

Cell proliferation was measured using the CellTiter 96 Aqueous One Solution cell proliferation assay (Promega, Madison, WI, USA). Cells were seeded in 96-well plates at a concentration of 10^4^ cells/well, allowed to adhere for 24 h, and subsequently exposed to different concentrated solutions as indicated below. Proliferation viability was measured according to the manufacturer's instructions after 48 h. The absorbance value at 490 nm was measured with an ELISA reader (BioTek, Vermont, USA). The stock solution of Huaier extract was diluted at final concentrations of 0, 2, 4, 8, and 16 mg/mL with complete 1640 medium. The viability ratio was calculated according to the following formula: The Viability Ratio = [(the absorbance of experimental group − the absorbance of blank group)/(the absorbance of untreated group − the absorbance of blank group)] × 100%.

### 2.4. Apoptosis Assay

Cell apoptosis was determined using the Annexin V-FITC apoptosis detection kit (Bio-science, Beijing, China). Briefly, 2 × 10^5^ cells were seeded into a 6-well plate. After 48 h exposure to different concentrations of Huaier aqueous extract as above, all the adherent cells were collected with 0.25% trypsin without EDTA, including the floating cells in the medium. Annexin V-FITC and propidium iodide (PI) were used for staining according to the manufacturer's instructions. The double-stained cells were subsequently analyzed by a FACSCanto flow cytometer (Becton-Dickinson, Mountain View, CA, USA). At least 10,000 cells were counted each time.

### 2.5. Hoechst 33258 Staining

Following treatment with Huaier extract at various concentrations for up to 48 h in a 6-well plate, cells were washed twice with PBS and fixed in 1 mL of 4% paraformaldehyde for 10 min at 4°C. After washing twice with PBS, cells were stained with 500 *μ*L Hoechst 33258 (Beyotime, Haimen, China) for 15 min at room temperature in the dark and then washed with PBS. Afterwards, the cells were mounted and examined under fluorescence microscopy (Olympus IX71, Tokyo, Japan). Apoptotic cells were identified by the condensation and fragmentation of their nucleus. The apoptotic ratio was obtained by the following calculation: The Apoptotic Ratio = apoptotic cell number/seeded cell number × 100%.

### 2.6. Cell Cycle Assay

Briefly, 2 × 10^5^ cells were seeded into a 6-well plate and starved in serum-free medium on the second day. After 12 h starvation, the cells were treated with gradient concentrations of Huaier solution for 48 h. The cells were then trypsinized, washed with cold PBS, and fixed overnight with 70% cold ethanol at 4°C. The next day, the fixed cells were centrifuged at 1200 g for 5 min and washed once with PBS. After that, the cells were suspended in PI/RNase staining buffer for 30 min in the dark according to the manufacturer's instructions of Cell Cycle Detection Kit (KeyGEN, Nanjing, China). Then, the DNA contents of the cells were analyzed in a FACScan flow cytometer (Becton Dickenson, San Jose, CA, USA). At least 10,000 cells were collected for each measurement.

### 2.7. Western Blot Analysis

The cells treated with Huaier extract or SP600125 (a JNK1/2 inhibitor, Beyotime, Haimen, China) for 48 h were washed twice with ice-cold PBS and lysed in ice-cold protein lysis buffer supplemented with 1% (v/v) protease inhibitor cocktail and PMSF. The lysates were centrifuged at 12,000 rpm for 10 min at 4°C. The suspension protein concentrations were determined using a BCA Protein Assay kit (Beyotime, Haimen, China) and were then denatured by boiling. Total proteins (25 *μ*g/lane) were resolved onto SDS-PAGE and transferred onto a PVDF membrane in a wet transfer system (Bio-Rad, USA) at 70 V at 4°C. For immunoblotting, the PVDF membrane was incubated with Tris-buffered saline plus Tween-20 (TBS-T) containing 5% nonfat milk for 1.5 h and then incubated with a specific primary antibody overnight at 4°C. Horseradish peroxidase- (HRP-) conjugated IgG was used as the secondary antibody and incubated for 2 h. Afterwards, reactive protein was detected using an enhanced chemiluminescence (ECl) commercial kit (Beyotime, Beijing, China). The results were recorded using the MicroChemi Bio-Imaging Systems (DNR Bio-Imaging Systems Ltd, Jerusalem, Israel) and Quantity One version 4.5.0 software (Bio-Rad, Hercules, CA, USA).

The primary antibodies used in this study were as follows: rabbit anti-pro-caspase 3 polyclonal antibody (Cat no. 19677-1-AP; Proteintech Group, Inc. Chicago, IL, USA), rabbit anti-cleaved-caspase 3 polyclonal antibody (Cat no. 25546-1-AP; Proteintech Group), rabbit anti-*β*-catenin polyclonal antibody (Cat no. 51067-2-AP; Proteintech Group), mouse anti-cyclin D1 monoclonal antibody (Cat no. 60186-1-Ig; Proteintech Group), mouse anti-GAPDH monoclonal antibody (Cat no. 60004-1-lg; Proteintech Group), rabbit anti-ERK polyclonal antibody (Cat no. 94; Santa Cruz Biotechnology, Inc., Santa Cruz, CA, USA), rabbit anti-p38MAPK polyclonal antibody (Cat no. 535; Santa Cruz Biotechnology), rabbit anti-JNK polyclonal antibody (Cat no. 571; Santa Cruz Biotechnology), mouse anti-pERK monoclonal antibody (Cat no. 7383; Santa Cruz Biotechnology), mouse anti- pp38MAPK monoclonal antibody (Cat no. 7973; Santa Cruz Biotechnology), mouse anti-pJNK monoclonal antibody (Cat no. 6254; Santa Cruz Biotechnology), and mouse anti-tubulin monoclonal antibody (Cat no. 0098; Cwbiotech, Beijing, China). The secondary antibodies included goat anti-rabbit IgG serum (1 : 40,000 dilution; Zhongshan Golden Bridge, Beijing, China) and goat anti-mouse IgG serum (1 : 40,000 dilution; Zhongshan Golden Bridge).

### 2.8. Statistical Analysis

All experiments were performed three times. Data were presented as means ± standard deviations (SD). The differences were analyzed using one-way ANOVA followed by the Student-Newman-Keuls test and all statistical analyses were performed using GraphPad Prism 5 software. Statistical differences are presented at probability levels of *P* < 0.05, *P* < 0.01, and *P* < 0.001.

## 3. Results

### 3.1. Huaier Extract Inhibits Cell Proliferative Viability of HepG2 and Bel-7402 Cells

**Figure 1:**
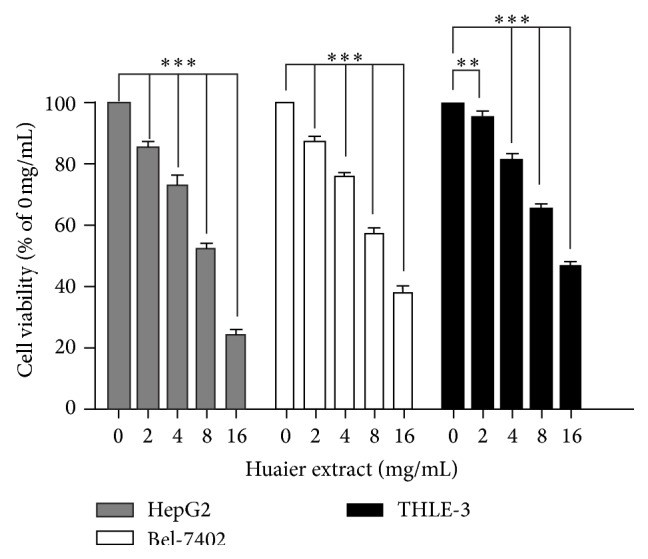
Effect of Huaier extract on the viability of HepG2, Bel-7402, and THLE-3 cells. HepG2, Bel-7402, and THLE-3 cells (10^4^ cells/well) were treated with various concentrations (0, 2, 4, 8, and 16 mg/mL) of Huaier extract for 48 h. Cell viability was determined by using an MTS assay. The results represent the means ± SD of 3 independent experiments. ^*∗∗*^ *P* < 0.01 and ^*∗∗∗*^ *P* < 0.001, compared with that of the untreated control, respectively (0 mg/mL).

To evaluate the proliferative effect of Huaier extract on HepG2 and Bel-7402 cells, we measured cell proliferative viability using the MTS assay after the cells were dose-dependently treated with Huaier extract for 48 h. As shown in Figure 1, Huaier extract significantly suppressed cell viability of both HepG2 and Bel-7402 cells in a dose-dependent manner with IC_50_ value of 7.6 and 10.6 mg/mL, respectively, after 48 h. But the IC_50_ value in the case of THLE-3 was 13.8 mg/mL, which means that the Huaier extract is less toxic to the normal liver cells than to HCC cells.

### 3.2. Huaier Extract Induces Cell Apoptosis in HepG2 and Bel-7402 Cells

**Figure 2:**
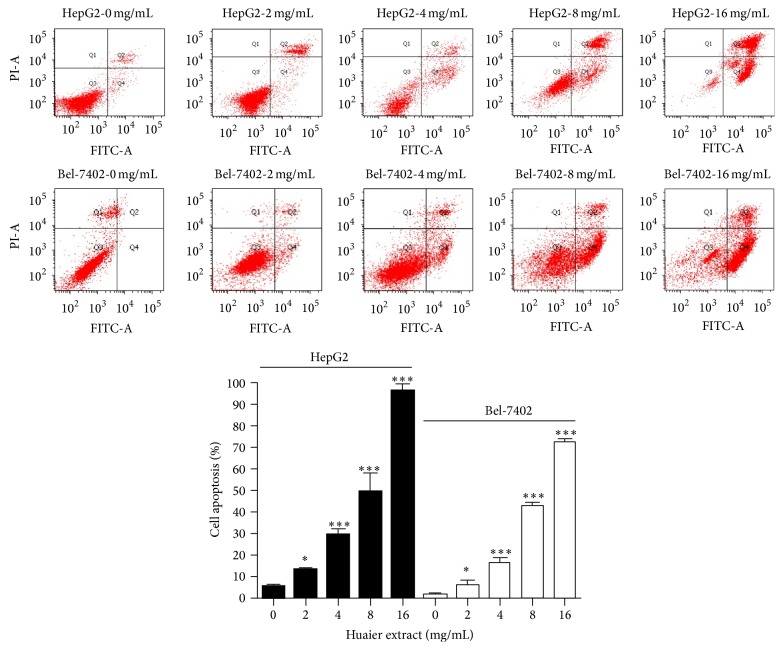
Effect of Huaier extract on the apoptosis of HepG2 and Bel-7402 cells. FCM analysis for apoptosis after treatment by Annexin V-FITC and PI staining on HCC cells with different doses of Huaier extract (0, 2, 4, 8, and 16 mg/mL) for 48 h. The ratios are expressed as the mean ratios ± SD in triplicate. ^*∗*^ *P* < 0.05 and ^*∗∗∗*^ *P* < 0.001, compared with that of the untreated control, respectively (0 mg/mL).

To demonstrate the apoptosis effect of Huaier extract, we used FCM analysis with Annexin V-FITC and PI double staining. After treatment with different doses of Huaier extract for 48 h, early apoptotic cells and late apoptotic cells were differentiated from viable or necrotic ones. In the control group, there were almost normal cells, rarely apoptotic cells, while in Huaier extract groups, the rates of apoptotic cells gradually increased along with increasing concentrations of Huaier extract. The rates of apoptosis in different Huaier extract (0, 2, 4, 8, and 16 mg/mL) groups were 5.50 ± 1.04%, 13.57 ± 0.58%, 29.40 ± 3.00%, 49.53 ± 8.50%, and 96.22 ± 3.06%, respectively, in HepG2 cells, and 1.5 ± 0.5%, 6.1 ± 2.1%, 16.6 ± 2%, 43 ± 1.5%, and 72.4 ± 1.6% respectively, in Bel-7402 cells (Figure 2).

### 3.3. Huaier Extract Induces Morphological Changes in HepG2 Cells

**Figure 3:**
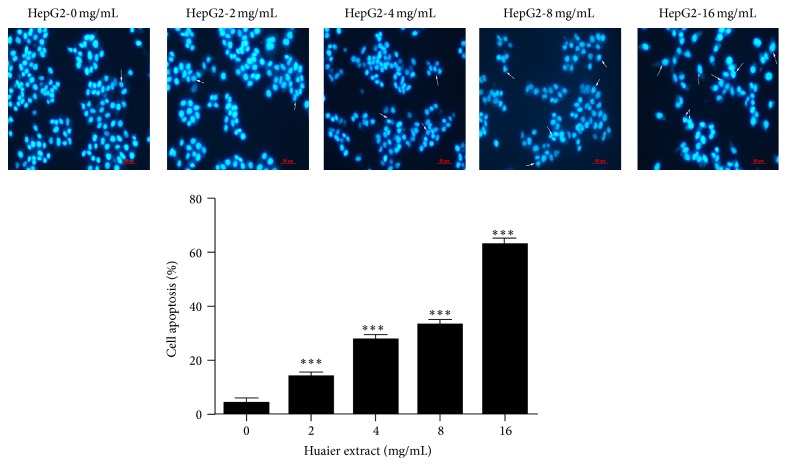
Effect of Huaier extract on the morphological changes of HepG2 cells. Hoechst 33258 staining was used to observe the apoptotic cells under a BX-60 fluorescence microscope (200x) after cells were treated with different doses of Huaier extract (0, 2, 4, 8, and 16 mg/mL) for 48 h. The arrow shows apoptotic cells. The values are expressed as the mean ratios ± SD from three independent experiments. ^*∗∗∗*^ *P* < 0.001, compared with that of the untreated control (0 mg/mL).

In addition, we verified the apoptotic effect of Huaier extract in HepG2 cells by morphological changes. After treatment with different doses of Huaier extract for 48 h, HepG2 cells were stained with Hoechst 33258. The normal cells in morphology are round and homogenous, while the morphological changes of cell apoptosis include cell shrinkage, nuclear condensation, and fragmentation. Fluorescence dye stains condense chromatin of apoptotic cells more brightly than chromatin of normal cells. The number of HepG2 cells adhering to the culture plates in Huaier extract treatment was greatly reduced compared to control group. The apoptotic morphological changes were observed in the Huaier extract-treated groups, whereas few apoptotic cells were found in the control group. The percentage of apoptotic cells in different Huaier extract (0, 2, 4, 8, and 16 mg/mL) groups was 4.27 ± 1.80%, 14.27 ± 1.20%, 27.60 ± 2.00%, 33.17 ± 1.90%, and 62.67 ± 2.40%, respectively, in HepG2 cells (Figure 3).

### 3.4. Huaier Extract Activates Caspase 3 and Induces the Expression of Cleaved Caspase 3 and Cleaved PARP in HepG2 and Bel-7402 Cells

**Figure 4:**
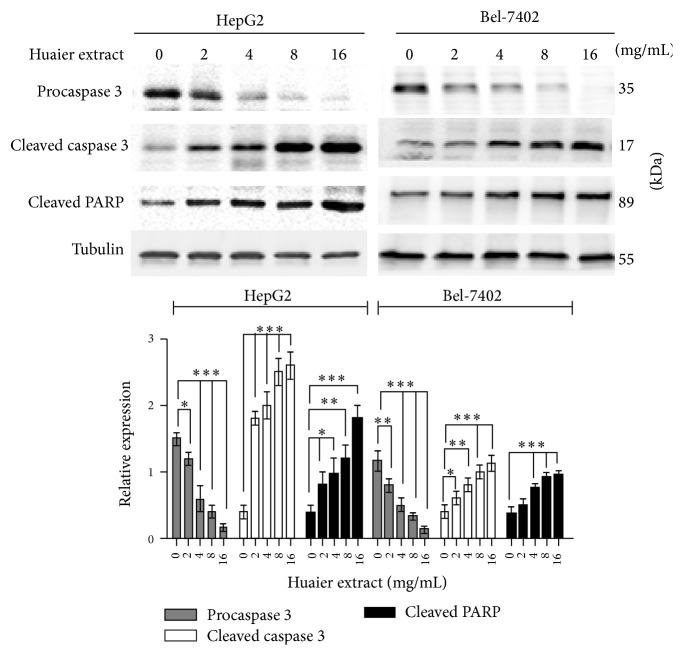
Effects of Huaier extract on expression of procaspase 3, cleaved caspase 3, and cleaved PARP in HCC cells. HCC cells were treated with various concentrations (0, 2, 4, 8, and 16 mg/mL) of Huaier extract for 48 h, and then cell lysates were subjected to Western blotting with procaspase 3, cleaved caspase 3, and cleaved PARP antibodies. The densitometric ratios were normalized to those of tubulin, and the results are expressed as the mean densitometric ratios ± SD in three independent experiments. ^*∗*^ *P* < 0.05, ^*∗∗*^ *P* < 0.01, and ^*∗∗∗*^ *P* < 0.001, compared with that of the untreated control, respectively (0 mg/mL).

To further confirm the apoptotic mechanisms of Huaier extract on HCC cells, we tested the expression of procaspase 3, cleaved caspase 3, and cleaved PARP with Western blot. Huaier extract activated the caspase 3, resulting in increased expression of cleaved caspase 3, cleaved PARP, and decreased expression of procaspase 3 (Figure 4).

### 3.5. Huaier Extract Induces Cells Arrest in S Phase of HepG2 and Bel-7402 Cells

**Figure 5:**
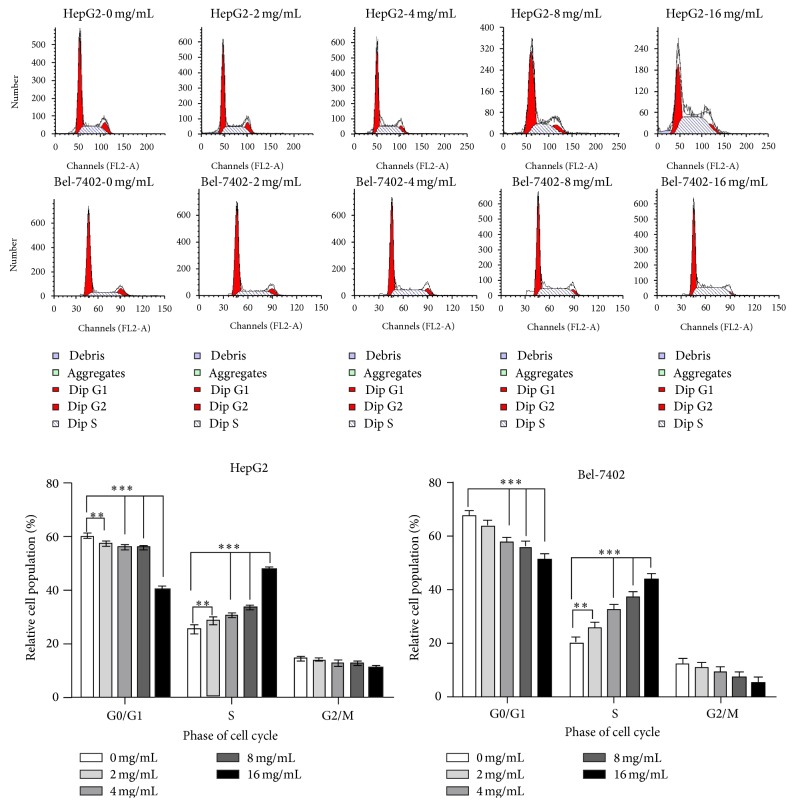
Effects of Huaier extract on the cell cycle of HCC cells by FCM. The cell cycle distributions in HepG2 and Bel-7402 cells were determined by PI staining and FCM analysis after being treated with different concentrations of Huaier extract (0, 2, 4, 8, and 16 mg/mL) for 48 h. The ratios are expressed as the mean ratios ± SD of three independent experiments. ^*∗∗*^ *P* < 0.01 and ^*∗∗∗*^ *P* < 0.001, compared with that of the untreated control, respectively (0 mg/mL).

To further investigate the effect of Huaier extract on the cell cycle, the cell cycle profiles of HepG2 and Bel-7402 cells were analyzed using flow cytometry. The cells were treated with Huaier extract at concentrations of 0, 2, 4, 8, and 16 mg/mL for 48 h and stained with PI. Huaier extract treatment resulted in a significant increase in the percentage of cells in the S phase and a significant decrease in the percentage of cells in the G0/G1 phase. The percentage of cells accumulated in the S phase were 25.31 ± 1.53%, 28.43 ± 1.25%, 30.48 ± 0.76%, 33.55 ± 0.81%, and 47.57 ± 0.87%, respectively, in HepG2 cells, and 20.25 ± 2.06%, 25.87 ± 2.06%, 32.43 ± 2.02, 37.11 ± 2.05, and 43.61 ± 2.33, respectively, in Bel-7402 cells. The accumulation of G0/G1 phase cells was maximal in the control group and declined with increasing concentrations of Huaier extract. The decrease in the number of G0/G1 phase cells was 60.36 ± 0.71%, 57.43 ± 0.95%, 56.13 ± 0.96%, 55.76 ± 0.54%, and 40.38 ± 0.88%, respectively, in HepG2 cells, and 67.4 ± 2.07, 63.62 ± 2.30, 57.5 ± 1.84, 55.66 ± 2.41, and 51.23 ± 2.10, respectively, in Bel-7402 cells (Figure 5). These results indicate that Huaier extract suppresses HCC cells proliferation by inducing S phase cell cycle arrest.

### 3.6. Huaier Extract Inhibits Cycle Related *β*-Catenin and Cyclin D1 Expression in HepG2 and Bel-7402 Cells

**Figure 6:**
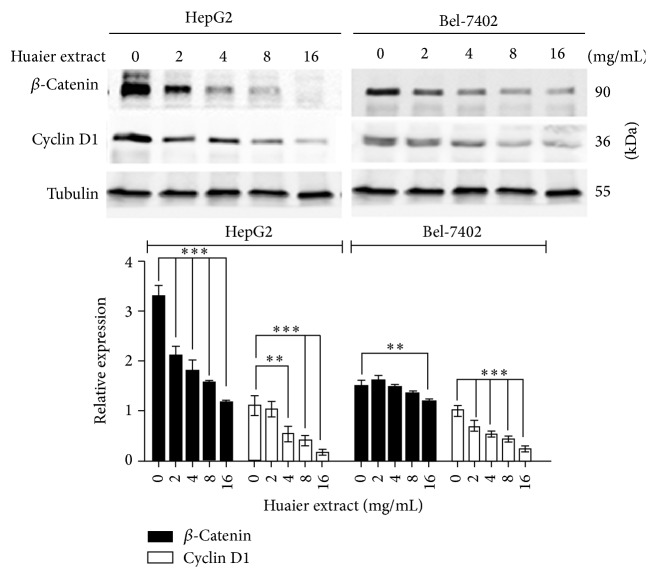
Effects of Huaier extract on expression of cycle related *β*-catenin and cyclin D1 in HCC cells. HCC cells were treated with various concentrations (0, 2, 4, 8, and 16 mg/mL) of Huaier extract for 48 h, and then cell lysates were subjected to Western blotting with *β*-catenin and cyclin D1 antibodies. The densitometric ratios were normalized to those of tubulin, and the results are expressed as the mean densitometric ratios ± SD in three independent experiments. ^*∗∗*^ *P* < 0.01 and ^*∗∗∗*^ *P* < 0.001, compared with that of the untreated control, respectively (0 mg/mL).

To elucidate the underlying mechanisms responsible for the proliferative inhibitory properties of Huaier extract on HepG2 and Bel-7402 cells, we detected changes of *β*-catenin and cyclin D1 expression by Western blotting and found that Huaier extract treatment significantly decreased the expression of *β*-catenin and cyclin D1 in a dose-dependent manner (Figure 6).

### 3.7. Huaier Extract Inhibits the Phosphorylation of MAPK in HCC Cells

**Figure 7:**
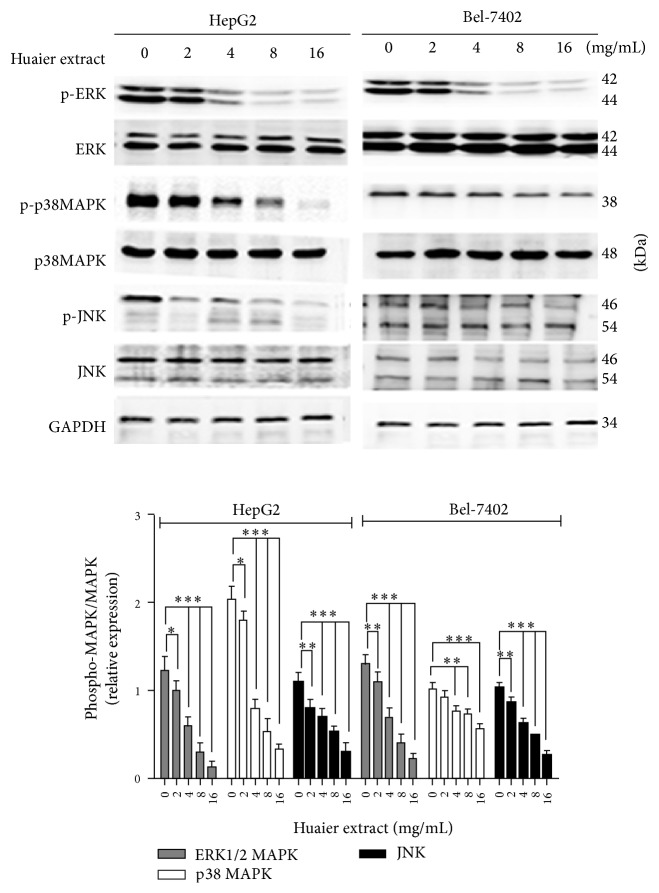
Effects of Huaier extract on expression of MAPK in HCC cells. HCC cells were treated with various concentrations (0, 2, 4, 8, and 16 mg/mL) of Huaier extract for 48 h, and then cell lysates were subjected to Western blotting using respective antibodies against MAPKs. Total ERK1/2, p38, JNK, and GAPDH were included as the loading controls, and the results are expressed as the mean densitometric ratios ± SD in three independent experiments. ^*∗*^ *P* < 0.05, ^*∗∗*^ *P* < 0.01, and ^*∗∗∗*^ *P* < 0.001, compared with that of the untreated control, respectively (0 mg/mL).

MAPK has been shown to be involved in the induction of cycle related *β*-catenin and cyclin D1 in many types of cancer [24]. Given that treatment with Huaier extract inhibited expression of *β*-catenin and cyclin D1, we attempted to determine the MAPK related protein expression by Huaier extract treatment in HCC cells. By treating HepG2 and Bel-7402 cells with various concentrations of Huaier extract, it was determined that there was a significant dose-dependent decrease in the phosphorylation of ERK1/2, p38, and JNK1/2 in HCC cells (Figure 7). This finding indicates that Huaier extract may suppress the expression of *β*-catenin and cyclin D1 by inactivating the MAPK pathways.

### 3.8. Huaier Extract Inhibits the Expression of *β*-Catenin and Cyclin D1 and Induces S Phase Arrest via the JNK Signaling Pathway in HCC Cells

**Figure 8:**
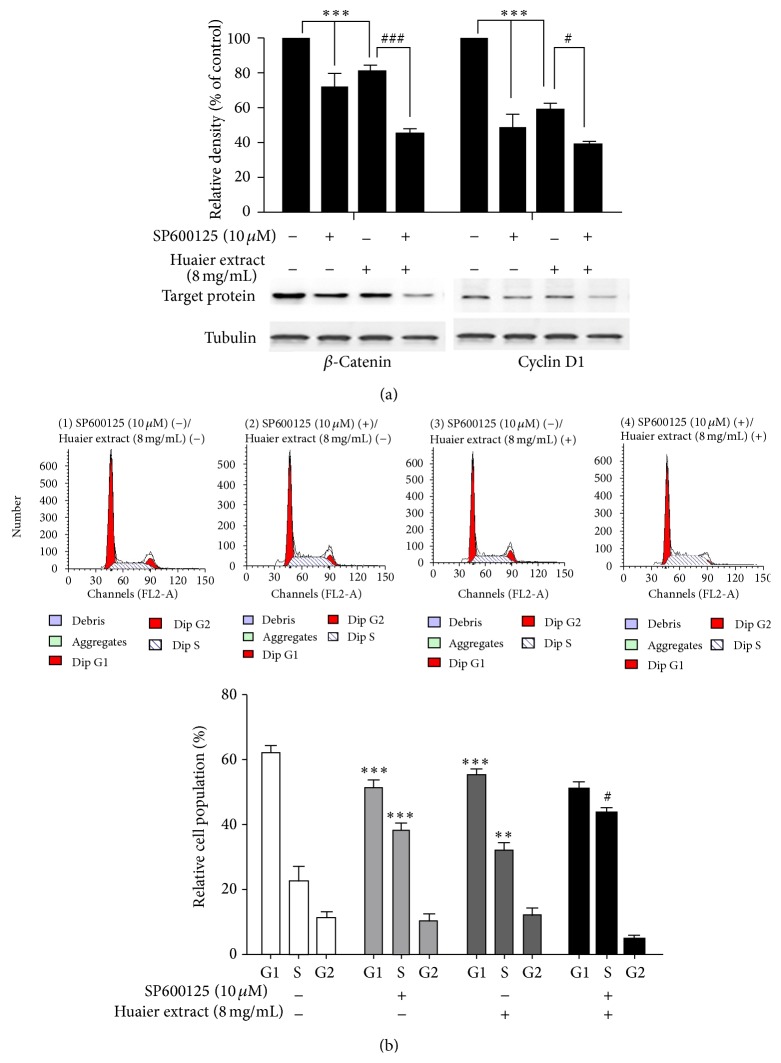
Effect of Huaier extract on the expression of *β*-catenin and cyclin D1, as well as cell cycle and inhibition after being treated with a JNK inhibitor (SP600125). (a) HepG2 cells were pretreated with SP600125 (10 *μ*M) for 1 h and then incubated in the presence or absence of Huaier extract (8 mg/mL) for 48 h. *β*-catenin and cyclin D1 protein was determined by using Western blotting. (b) Cells were also assessed by cell cycle. Data are presented as the means ± SD of at least three independent experiments. ^*∗∗*^ *P* < 0.01, ^*∗∗∗*^ *P* < 0.001, untreated cells versus SP600125 or Huaier extract; ^#^ *P* < 0.05, ^###^ *P* < 0.001 Huaier extract versus SP600125 plus Huaier extract.

To further investigate whether JNK plays a role in reducing the expression of *β*-catenin and cyclin D1, HepG2 cells were pretreated with SP600125 (10 *μ*M) for 1 h and then incubated with Huaier extract (8 mg/mL) for 48 h. Using Western blotting assay, it was found that treatment with either Huaier extract (8 mg/mL) or SP600125 (10 *μ*M) reduced *β*-catenin protein levels by 19% and 28.4% and cyclin D1 protein levels by 41% and 51.7%, respectively, and that combination treatment reduced *β*-catenin and cyclin D1 protein levels by 54.7% and 60.7%. These data suggest that Huaier suppresses the expression of *β*-catenin and cyclin D1 through the downregulation of JNK signaling in HepG2 cells. Moreover, in a functional assay of cycle inhibitory properties, SP600125 also increased the percentage of HepG2 cells in the S phase, decreased the percentage of cells in the G0/G1 phase, and facilitated the Huaier extract induced S phase arrest in HepG2 cells (Figure 8). All these results indicate that Huaier extract suppresses HCC cells growth by inducing S phase cell cycle arrest through JNK signaling pathway.

## 4. Discussion 

Huaier electuary ointment, of which the active ingredient is extracted from fungi of Huaier, has been used in clinic for the treatment of hepatocellular carcinoma with satisfactory results [26]. It has been showed that Huaier extract inhibits the proliferation and tumor angiogenesis and induces apoptosis of hepatocellular carcinoma cells [17]. Additionally, Huaier extract has also been indicated to suppress the hepatocellular carcinoma biological activities of adhesion, migration, and invasion related to epithelial–mesenchymal transition (EMT) [16]. All these findings provide a certain rationale for therapeutic properties of Huaier extract in clinical applications of hepatocellular carcinoma. However, the potential molecular mechanisms are still elusive and require further validation.

In the current study, the results of MTS assay demonstrated that Huaier extract significantly attenuated the proliferation of HCC cells in a dose-dependent manner. To our knowledge, the inhibition of cell proliferation was involved in apoptosis and block of cell cycle progression. It has been reported that Huaier extract induced melanoma cells and breast cancer cells apoptosis via the increased expression of P53 and the modulation of Bcl-2/BAX protein expression [13, 18]. Moreover, Huaier extract also caused apoptosis in lung cancer cells via a miR-26b-5p-EZH2-mediated approach [11]. Ren et al. reported that Huaier extract induced HepG2 cells apoptosis by the detection of flow cytometry [17]. However, little is known on the exact apoptosis mechanism of Huaier extract on HCC cells. Of note, our data ascertained that Huaier extract induces the onset of apoptosis by activating caspase 3, increasing the expression of cleaved caspase 3 and cleaved PARP in HCC cells.

Eukaryotic cell proliferation is primarily regulated by the cell cycle, which consists of four phases: the G1 phase, the S phase, the G2 phase, and the M phase [27]. It is well established that the loss of key cell cycle checkpoints is a hallmark of cancer cells, which leads to abnormal proliferation and facilitates oncogenic transformation [28]. The G1/S transition is one of the two predominant checkpoints of the cell cycle and is responsible for the initiation and completion of DNA replication. The majority of studies have reported perturbation of the S/G2 phase transition with a decrease of cells in the G0/G1 phase of the cell cycle and an increase of cells in the S phase [28, 29]. In the present study, FACS analysis with PI staining revealed that the percentage proportion was increased in the S phase cells and reduced in the G0/G1 phase cells following Huaier extract treatment in a dose-dependent manner, indicating that the inhibitory effect of Huaier extract on HCC cell proliferation is mediated by S phase cell cycle arrest.

In the current study, the expression of the important cycle regulatory protein, cyclin D1, and its upstream effector *β*-catenin were analyzed following the treatment of HepG2 and Bel-7402 cells with Huaier extract and the results were consistent with previous observations that S phase arrest is accompanied by the decreased expression of cyclin D1 and *β*-catenin [30, 31]. The modifications of these cell cycle-associated proteins induced by Huaier extract appear to block the cell progression through the S phase.

Activated MAPK pathways play a central role in HCC proliferation and cell cycle development [32, 33]. However, there are no reports and literatures of the relevant studies illustrating the relationship between Huaier extract and MAPK pathways on the proliferation of HCC. Therefore, we investigated the effect of Huaier extract on MAPK pathways in HCC cells and found that Huaier extract significantly downregulated the phosphorylation of ERK, p38MAPK, and JNK in HepG2 and Bel-7402 cells in a dose-dependent manner. What is more, we further investigated the role of JNK on cell cycle distribution and cycle related protein expression of cyclin D1 and *β*-catenin. Noticeably, treatment with SP600125 obviously decreased expression of cyclin D1 and *β*-catenin and also caused a significant increase in the percentage of cells in S phase and decrease in the percentage of cells in the G0/G1 phase, which was consistent with the treatment of Huaier extract on cells. All these results demonstrate that Huaier extract induces hepatocellular carcinoma cells arrest in S phase via JNK signaling pathway.

Overall, our findings verify that Huaier extract causes HCC cell apoptosis and induces hepatocellular carcinoma cells arrest in S phase via JNK signaling pathway, which advances our understanding on the molecular mechanisms of Huaier extract in hepatocarcinoma management. The clinical treatment for liver cancer is difficult due to its malignant biological characteristics such as invasion, metastasis, and malignant proliferation. In this study, we explored the antiproliferative mechanisms of Huaier extract on HCC cells, providing a novel prospect for liver cancer treatment. Huaier extract serves as a promising therapeutic drug for liver cancer, not only for a potent apoptosis inducing, antiangiogenic, and anti-invasive agent, but also for the block role in cell cycle progression.
